# Impact of Current and Temperature on Extremely Low Loading Epoxy-CNT Conductive Composites

**DOI:** 10.3390/polym12040867

**Published:** 2020-04-10

**Authors:** Brian Earp, Jonathan Phillips, Dragoslav Grbovic, Stephen Vidmar, Matthew Porter, Claudia C. Luhrs

**Affiliations:** 1 Department of Mechanical and Aerospace Engineering, Naval Postgraduate School, Monterey, CA 93943, USA; ccluhrs@nps.edu; 2 Energy Academic Group, Naval Postgraduate School, Monterey, CA 93943, USA; jphillip@nps.edu; 3 Department of Physics, Naval Postgraduate School, Monterey, CA 93943, USA; dgrbovic@nps.edu; 4 Naval Research Enterprise Internship Program, Naval Postgraduate School, Monterey, CA 93943, USA; stephen.vidmar5@gmail.com; 5 Department of Electrical Engineering, Naval Postgraduate School, Monterey, CA 93943, USA; maporter@nps.edu

**Keywords:** carbon nanotubes, epoxy-CNT composites, electrical resistivity

## Abstract

Carbon nanotube (CNT) conductive composites have attracted significant attention for their potential use in applications such as electrostatic dissipation and/or electromagnetic interference shielding. The focus of this work is to evaluate resistivity trends of extremely low loading (<0.1 wt%) epoxy-CNT composites that lack a connected CNT network, but still present electrical conductivity values appropriate for those uses. The impact of current, temperature, and cycle life on electrical properties are here identified and tied to possible performance limits. At extremely low loadings, the CNT content is not sufficient to form a completely interconnected grid, thus, electrons must travel through insulating media. While still in the semi-conductor range, resistivity values are observed to decrease with increasing direct current and demonstrate a non-ohmic behavior. CNT epoxy composites were subjected to elevated currents and/or temperatures over diverse periods of time to examine impacts on resistivity. Microstructural analyses of composite samples were conducted to observe signs of damage for specimens taken to extreme temperatures/currents. An understanding of the electrical conductivity characteristics of extremely low loading epoxy-CNT composites and their failure mechanisms will aid in understanding risks associated with their use in challenging environments that may include high temperatures, high currents, and/or high frequencies.

## 1. Introduction

The use of carbon nanotubes (CNT) as filler material in polymeric composites has shown great potential for applications such as electrostatic discharge (ESD) and electromagnetic interference (EMI) prevention [1,2,3,4,5,6,7,8,9,10]. In particular, the CNT composites’ electrical conductivities reported by various research groups provide the basis for their broad applicability in space systems [1,2,3,4,5,6,9,10]. While development of EMI prevention devices has been focused on metallic meshes or metallic-resin composites [6,8,11,12] these devices are prone to significant disadvantages in terms of weight and cost [6,8,12,13]. The use of CNT composites for EMI prevention offers a potential solution that helps mitigate the weight and cost issues of metallic-based devices while still having the necessary properties that are critical for operations in challenging space environments.

While polymeric matrices are typically highly insulating, the dispersion of small amounts of electrically conductive nanomaterials of large aspect ratios, such as CNTs, can drastically improve electrical conductivity to levels that can support use for ESD or EMI shielding [2,5,6,7,9,10,11,13,14]. In order for conductive composite materials to be used in EMI and ESD applications, they must possess resistivity values within or below the ranges of 10–10^6^ and 10^6^–10^11^ Ohm·cm, respectively [14]. Additionally, the ability to tailor or modify CNT concentrations and/or composite synthesis and fabrication processes can support the rapid change of electrical properties. In the case of CNT composites, many parameters, such as CNT concentration, dispersion methods, curing time/temperature, type of CNTs, etc., can significantly impact the conductive properties of epoxy-CNT composites [15,16,17,18,19]. Direct current (DC) and alternating current (AC) electrical testing of epoxy-CNT composites shows that electrically conductive levels, necessary for use in ESD and EMI applications, can be easily achieved. Remarkably, even at extremely low CNT loadings (<0.1 wt%), epoxy-CNT composites meet these conductivity values at ambient conditions [15,16,19,20,21]. Prior observations indicate that the order of magnitude of the electrically conductive behavior, and the microstructure associated with such, are a function of CNT content; high to low loadings (>0.1 wt%) present connected CNT networks and resistivities in the order of ~1 to 10 Ohm·cm, while extremely low CNT loading (<0.1 wt%) exhibit unconnected CNT strands and resistivities in the order of 10^2^ to 10^4^ Ohm·cm [15].

The present work aims to offer a better understanding of the electrical behavior for the less examined compositional range: CNT-epoxy composites with extremely low CNT loadings (<0.1 wt%). Since it is not known if those composites meet the conductivity requirements at all the conditions that could be anticipated for space environments, this manuscript explores some of the variables that are believed could drastically affect their electrical behavior. The trends observed will help determine the suitability of these materials for uses under extreme conditions and will aid future efforts to tailor the desired properties in epoxy-CNT composite architectures. The manuscript addresses the influence of (i) current, (ii) temperature, and (iii) cycle life/aging in electrical properties and supplements that data with (iv) an analysis of which conditions will promote changes and failure, along with (v) an examination of the mechanisms that could explain the experimental observations.

## 2. Materials and Methods

### 2.1. Materials

All CNTs (Miralon Pulp^®^) used in the experimental work presented herein were obtained from Nanocomp Technologies Incorporated (Merrimack, NH, USA, Parent organization: Huntsman Corporation). The multi-wall, non-functionalized, CNTs were produced using a chemical vapor deposition process using iron as a catalyst to create large sheets of CNTs. The sheet resistivity has been reported to be 5 × 10^−4^ Ohm·cm. The sheets were then fragmented into bundles of approximately 0.05 mm in diameter and 1 mm in length using a Hollander Beater and an industrial burr mill [22]. The CNTs were received as bundles of highly entangled CNTs (pulp). The individual CNT diameters varied between 5 and 15 nm.

In order to generate the epoxy-CNT composites, LOCTITE EA9396 AERO epoxy paste adhesive (Hysol EA9396, Henkel Corporation, Dusseldorf, Germany) was used as the matrix. EA9396 is a two-part epoxy that is mixed at a ratio of 100 Part A to 30 Part B. All samples studied herein were cured for one hour at 66 degrees Celsius [23]. Custom prefabricated electrical measurement boards were used as the support for the generated epoxy-CNT composites and to conduct electrical testing.

### 2.2. Characterization and Measurement Tools

Scanning electron microscopy (SEM) observation of the CNTs and epoxy-CNT composites microstructures was performed using a Zeiss Neon 40 (Carl Zeiss Inc., Thornwood, NY, USA) field emission SEM operating between 1 and 20 kV. The instrument is coupled with an INCA Energy 250 Energy Dispersive X-ray microanalysis system with analytical drift detector. DC electrical resistivity measurements were conducted using a 2400 Keithley Source Meter (Beaverton, OR, USA) or Harrison 6110A DC Power Supply (Palo Alto, CA, USA) and a multimeter. CNT pulp measurements were performed using a Lucas Labs Pro4 four-point probe (Gilroy, CA, USA). AC electrical measurements were performed using a QuadTech 7600 Precision LCR Meter Model B (Maynard, MA, USA)) and associated LABVIEW software for data collection. Thermal images taken during electrical measurements were generated using a FLIR ETS320 thermal imaging system (Wilsonville, OR, USA). Heating of samples was performed using a VWR International, LLC hot plate/stirrer (Radnor, PA, USA).

### 2.3. Epoxy-CNT Composite Fabrication Process

CNTs and EA9396 were combined to form epoxy-CNT composites based on methodology from previous studies [15]. Requisite amounts of Part A—EA9396 epoxy resin and CNTs were measured and added to either a Max 10 or PP50 Flaktek mixing cup (depending on sample size) [24]. The amount of CNTs added to the Part A resin was based on desired weight percentage of CNTs using the total of Part A, Part B, and CNTs. Samples were mixed using a dual asymmetric centrifugal mixing process employing a FlackTek, Inc. Speed Mixer Model DAC150.1 FV2-K (Landrum, SC, USA) [25]. All samples analyzed in this study were mixed using an initial two cycles at low speed followed by a cooling period to prevent excessive heat buildup. Following cooling, the samples were mixed at three higher speeds for one minute with cooling periods between each mixing cycle. After completion of mixing Part A and CNTs, Part B (hardener) was added and hand mixed for five minutes.

Strips of 10 mm wide adhesive tape were applied to eight different locations on an electric testing board, each location connected to four-point metal terminals. Each of the eight testing locations consisted of a 10 mm × 10 mm testing area with thickness of the testing area determined by adhesive tape thickness. Following the mixing procedure, the epoxy-CNT mixture was spread over the testing locations by placing some of the mixture between the adhesive tape and then using a glass slide to level the sample surface before curing. Samples were cured in a Lindberg test furnace for one hour at 66 degrees Celsius.

### 2.4. DC Electrical Resistivity Measurements

DC current, ranging between 5 and 500 μA, was applied for most DC measurements. However, currents up to ~7000 μA were used for some parts of the analysis as described below. A test box, to allow the four-point testing board to be inserted for ease of measurements, a Keithley Source Meter, to provide current to the two outer electrodes, and a multimeter, to measure voltage differential across the two inner electrodes, were used for measurements up to 500 μA. For higher currents, the test box was not used because of a conflicting setup process and instead, the four-point testing board was connected directly to a Harrison 6110A DC Power Supply to provide current. To ensure the test box was not impacting the measurements, measurements were taken both with and without the test box when using the Keithley Source Meter with no significant differences observed. Minor differences in voltage differential readings for the same applied current did exist between the Keithley Source Meter and the Harrison 6110A DC Power Supply, however, these were not substantial enough to impact the comparison of the results.

The majority of the electrical resistivity measurements were taken by starting at the lowest applied current (5 μA) and increasing current to the desired upper value (500 μA) to minimize the temperature increases when conducting measurements. During higher current measurements, temperature changes were monitored using the FLIR thermal imaging device. In addition to DC electrical measurements with increased current, measurements at elevated temperatures, while maintaining a constant DC current of 500 μA, were performed by heating the four-point test board using a hot plate to raise the temperature. Voltage differentials were measured, and the temperatures were recorded while maintaining the current constant. All DC measurements supported the determination of a resistance value, that along with sample dimensions, yielded electrical resistivity values. The resistivity measurement and calculation process was the same as the method used in prior work [15].

### 2.5. AC Electrical Resistivity Measurements

AC electrical measurements were performed on epoxy-CNT samples using a Quadtech inductance, capacitance, resistance (LCR) meter with a design frequency range of 10 Hz to 2 MHz. The same text box from lower current (5 μA to 500 μA) DC electrical resistivity measurements was used for AC measurements. Input parameters for the Quadtech LCR are as follows: sweep parameter (frequency); signal type (voltage); frequency range (10 Hz to 2 MHz or 10 Hz to 1 kHz or 1 kHz to 2 MHz); voltage (0.5 V); current (250 μA); step size (200)—max allowed by instrument; accuracy (basic-fast); range hold (auto); and averages (10). Selection of step size, accuracy, and averages was based on obtaining a maximum of data points allowed by Quadtech LCR for a given frequency range with manufacturer stated accuracy of 0.5% and averaging that limited each individual measurement cycle to less than approximately 10 min.

## 3. Results and Discussion

### 3.1. Impact of Current on Composites Resistivity

#### 3.1.1. DC Measurements

**Figure 1:**
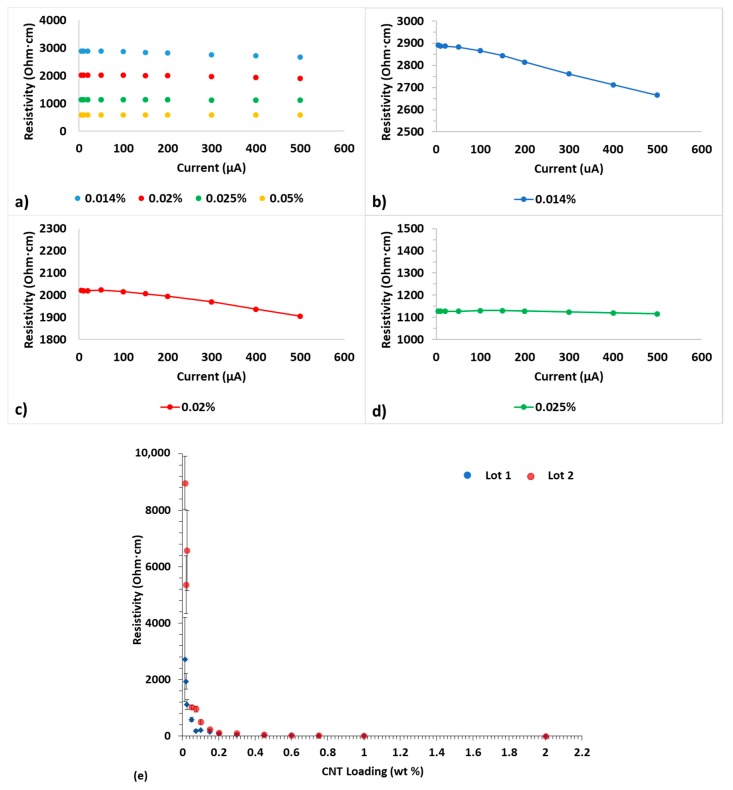
DC current effect on resistivity from 5 to 500 μA—(**a**) comparison for various CNT loadings, (**b**) 0.014 wt% CNT loading, (**c**) 0.020 wt% CNT loading, (**d**) 0.025 wt% CNT loading. Graph (**e**) presents the resistivity values encountered for diverse CNT loadings from [15], performed at 400 μA, for comparison.

In previous work on DC measurements of epoxy-CNT composites, specifically EA9396 epoxy with Nancomp CNTs, it was clearly shown that resistivity values decreased as the concentration/loading of CNTs increased [15]. It was also reported that DC resistivity, as a function of current, is sensitive to CNT loading. For CNT loading above ~0.1 wt% no significant current dependence of resistivity was found over the current range of 5 to 500 μA. In contrast, preliminary work for extremely low loadings (0.014 wt% CNT) indicated a ~10% reduction in resistivity when increasing current over the same range [15]. In current work, those trends were verified as seen in Figure 1a. The epoxy-CNT samples show a clear reduction in resistivity as the CNT content increases from 0.014 wt% to 0.05 wt%, however, only those with CNT loadings at or below ~0.025 wt% present a reduction in resistivity as the current is increased from 5 to 500 μA as seen in Figure 1b–d. The most noticeable decrease in resistivity (~10%), with increasing DC current occurred in the sample with the lowest CNT concentration (0.014 wt% CNT), while there was minimal decrease in resistivity (~5%) at a loading of 0.025 wt% CNT and almost no decrease at 0.05 wt% CNT. Based on the measured resistivity values, typically between 10^3^ Ohm·cm and 10^4^ Ohm·cm for 0.014 wt% samples, the epoxy-CNT composites studied herein fall within the typical resistivity range of semi-conductors [14,26,27].

To ensure that the lowering resistivity was not simply an artifact of the testing board for higher resistance samples, 22 kOhm and 55 kOhm resistors were affixed to a measurement board and resistance values were measured using the same equipment. Resistance values for both resistors over the 5 to 500 μA range remained within 0.25% of their respective averages across all measured currents. That is, unlike extremely low loading epoxy-CNT composites, for standard resistors no link to current or temperature was observed at these applied currents. Moreover, epoxy composites studied herein with CNT loadings above 0.025 wt% did not show either a current dependency or a rise in temperature when diverse currents were employed for their electrical properties’ determination. It is worth noting that the resistivity of the CNT bundles, with no epoxy added, was measured to be 0.056 ± 0.01 Ohm·cm. Such value was obtained with a four-point probe, after dispersing the CNTs in ethanol on top of a glass slide and allowing the solvent to evaporate. This value of resistivity is believed to be much higher than that of the individual tubes or bundles because of junction (interparticle) resistance contributions.

#### 3.1.2. AC Measurements

**Figure 2:**
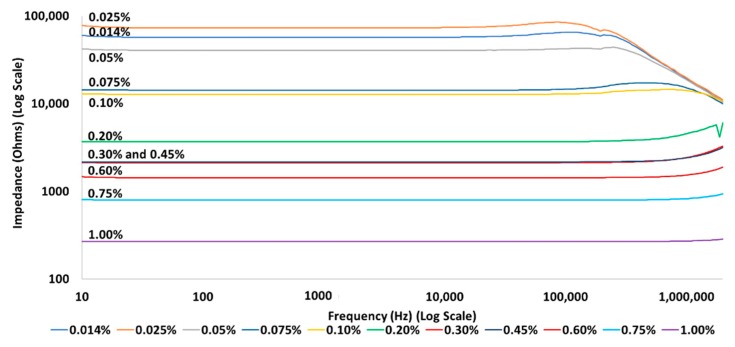
Log plot of impedance vs. frequency for various CNT loadings.

AC measurements were taken for various CNT loadings from 0.014 wt% to 1.0 wt% to examine any changes in impedance/phase angle. For all CNT loadings impedance values at the lower frequencies (below ~50 kHz) were flat and correlated well with DC resistance values (Figure 2); however, at high frequencies (>100 kHz) the impedance trends were found to be a function of CNT loading. At high frequency the impedance decreases as the frequency increases for samples with extremely low CNT loading (0.1 wt% and below), whereas for higher CNT loadings (e.g., 1 wt%) a constant or slightly increasing (0.2–0.75 wt%) impedance was observed with increasing frequency. The slight latter upshift was assessed to be an inductive effect because of the wiring/cabling used in the measurements. The decrease in impedance observed for the extremely low loading samples was not found to be associated with any spurious signal as similar trends were observed for multiple specimens with varied sample dimensions.

It is worth noting that, unlike low loading samples, conductivity is not a linear function of loading for extremely low loading samples. For example, in this study it was found that the sample with 0.014 wt% CNT loading had higher conductivity than the sample with 0.025 wt% CNT loading. A similar phenomenon was observed in DC measurements in previous reports, when amounts of CNT in a non-conductive matrix are extremely low, the variability between samples of similar net loading becomes very large [15]. This is best understood by a comparison of equivalent circuits for low loading (0.2% and above) and extremely low loading (0.1% and below) samples. In low loading samples, there will be local variations in CNT concentration, high in some areas, low in other areas. However, overall the “connectivity”/conductivity for the whole sample will represent an average. That is, the composite can be modelled with an equivalent circuit consisting of many resistors in series and parallel. For such a large network, gross properties will average out. It is expected, and reported, that all samples of the same low loading will show almost identical average behavior. In contrast, for extremely low loading samples, variations in local CNT concentration are not “averaged out.” The number of resistors/capacitors in the equivalent circuit is far smaller than that in the low loading samples; change a few and the gross behavior of the sample is changed. In sum, the data make it clear that the trend of impedance at high frequency is impacted by the CNT loading. The data also show a distinct “break,” by order of magnitude between loadings of 0.1% and below and those of 0.2% and above, and suggest that composites with extremely low CNT loading might have potential for use as high-pass filters.

The data for AC conductivity measurements observed over a wide frequency range are comparable to the admittance and conductivity measurements published for SWNTs [20] which showed an increase in admittance for a 0.025 wt% sample. Data presented by Backes et al. for an epoxy resin (Araldite LY1316 with Aradur HY1208 hardener) and MWCNT composite show an increase in electrical conductivity (decrease in resistivity) as frequency is increased for a 0.05 wt% CNT sample, a smaller increase in conductivity for a 0.1 wt% sample, and essentially no change for a 0.2 wt% and 0.3 wt% sample [28]. Similar work by Sandler et al. with epoxy resin (Araldite LY556 with Araldite HY932 hardener) and MWCNTs showed increasing conductivity with increasing frequency for 0.001 wt% and 0.0025 wt% samples, however, 0.005 wt% samples and higher CNT concentrations exhibited nearly linear conductivity values up to a frequency of 100 kHz [21]. The data presented in Figure 2 is comparable to the Sandler et al. data up to about 10 kHz, however, it should be noted that electrical conductivity values for the bare epoxy used in the two presented articles are much higher (~5 to 6 orders of magnitude) than the reported conductivity (converted from resistivity stated by manufacturer) of ~4.5 × 10^−^^14^ Ohm·cm [23] for the epoxy used in this work.

### 3.2. Impact of Temperature on Composites Resistivity

Even though measurements at different DC currents described in Section 3.1.1. were taken in a manner to minimize the role that other variables played in the electrical data, temperature changes due to Joule heating were observed in extremely low loading samples. These changes were noted to correlate with increases in current and concomitant decreases in resistivity. Thus, experiments were carried out to consistently evaluate the effect of temperature, both dependent and independent of the changes in current, on the resistivity of the extremely low loading epoxy CNT composites. Temperature changes generated in two fashions were studied: (a) Induced by DC currents, and (b) imposed temperature increase using a hot plate at a constant current.

#### 3.2.1. Temperature Increase Driven by DC Current

**Figure 3:**
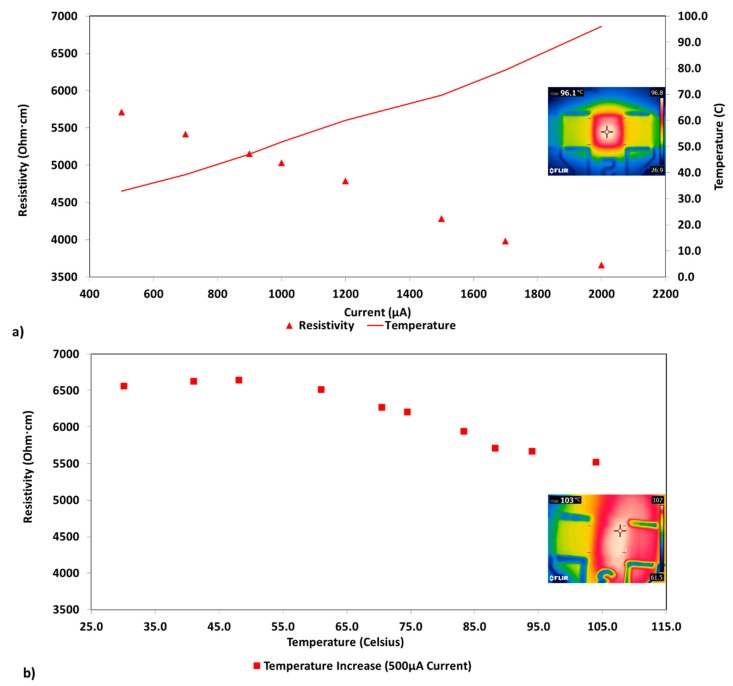
Temperature impacts on resistivity. (**a**) Increasing current from 500 μA to 2000 μA with a 0.014 wt% CNT sample. (**b**) Increasing temperature from room temperature to greater than 100 degrees Celsius with a 0.014 wt% CNT sample at a constant current of 500 μA.

As discussed in prior work [15] for various CNT lots, and verified with the current study, conductivity is directly proportional to current. However, in samples with extremely low CNT loadings (0.014 wt% CNT is used here as example) another effect is detected; as current is increased, temperature also rises. This provokes a question: Does low frequency/DC conductivity increase in extremely low loading samples with increasing current result primarily from temperature increase? As shown in Figure 3a after increasing the current, the resistivity at 2000 μA was markedly lower (~3660 Ohm·cm) than when measured at 500 μA (~5710 Ohm·cm). Additionally, the sample temperature increased from ~33 to ~96 degrees Celsius over the applied current range. Also, it is worth noting that there is variability in the actual resistivity values observed between different specimens, however, the current–temperature trends reported herein are similar for all samples.

#### 3.2.2. Imposed Temperature Alteration Using a Hot Plate

Based on the observed change in resistivity at increased current/temperature a second method was used to conduct an examination of the impact of temperature effects while maintaining a constant applied DC current. Specifically, a hot plate was used to increase the test board temperature from room temperature to above 100 degrees Celsius while maintaining a constant current of 500 μA. The resultant values of resistivity vs. temperature can be seen in Figure 3b. A similar reduction in resistivity than the one seen with the increase of current in Figure 3a can be seen in Figure 3b when only a hot plate was used to impose the temperature change. Increasing temperature from ~30 to 104 degrees Celsius results in a resistivity change from ~6560 to ~5500 Ohm·cm, an approximately 15% reduction.

The main differences between temperature changes driven by current and those imposed by a hot plate indicate that increasing current, which causes a concomitant increase in local sample temperatures, results in a more significant change in resistivity than simply increasing sample temperature. The sample subjected to a current increase from 500 to 2000 μA, showed a resistivity drop from ~5710 Ohm·cm at 32.9 degrees Celsius and 500 μA to ~3660 Ohm·cm at 96.0 degrees Celsius and 2000 μA which is roughly a 35% decrease. The sample subjected to a temperature increase via the hot plate alone showed a resistivity drop from ~6560 Ohm·cm at 30.1 degrees Celsius to ~5670 Ohm·cm at 94 degrees Celsius which is roughly a 15% decrease over roughly the same temperature range.

It is postulated that the trends in conductivity with temperature from two method of heating is consistent with a single model: conductivity in extremely low loading samples directly correlates with temperature along current paths. Each method measures the temperature at the surface, yet in neither case it is an accurate measure of the temperature along the conductive paths. The fact that there is not perfect quantitative agreement for the two heating methods is expected; in one case heat is transferred from the hot plate through the testing board and composite to the sample surface and in the second case the measured surface temperature does not represent the internal temperature that is induced along the current path where CNTs are located. Hence, conductivity is a direct function of the temperature along the conductive paths.

Experiments indicating resistivity decreases with temperature are difficult to explain with certainty. In typical semiconductors, both intrinsic and extrinsic, resistivity tends to decrease with increasing temperature [29]; whereas, in a typical conductor, resistivity tends to increase with increasing temperature [26,27]. Based on this study, the extremely low loading epoxy-CNT composites exhibit resistivity values in the semi-conductor range and the decrease in resistivity seen with increasing current for the extremely low loading epoxy-CNT composites could, to a great extent, be attributed to temperature changes, that is, they present a negative temperature coefficient of resistance.

Part of the novelty of the study presented herein is the validation that the mechanisms of electrical conduction at elevated temperatures are drastically different between CNT epoxy composites with loadings 0.1–0.2 wt% and above, and those being the focus of this work with extremely low loading (<~0.1 wt%). A review of literature in the field indicates that although the exact loadings are slightly different, other published work agrees with the trends observed, showing lowering resistivity values as temperature is increased for composites with very low CNT contents. An example is found in the properties reported for epoxy-CNT composites containing 0.05 wt%, 0.1 wt%, 0.3 wt%, and 0.5 wt% CNTs presented by Shen et al. [30]. Work by Sanli et al. examined the impact of temperature for thin film epoxy-CNT composites (MWCNTs in epoxy resin L20 with EPH-161 hardener) using electrochemical impedance spectroscopy for different CNT wt% finding that a 0.5 wt% sample showed an 11.40% decrease in resistance over a temperature range of 20 to 80 degrees Celsius [31]. The reported percentage decrease in resistance in the later, tested over a roughly similar temperature range as in Figure 3b, is comparable to our observed percentage change in resistivity. While temperatures below ambient were not analyzed for the current study, lower resistivity values at room temperature when compared to those at 77 K, were also reported for polyester-CNT composites with loadings of 0.1, 0.3, and 0.5 wt% [32].

It is believed that the exact CNT loading at which the electrical behavior of the composite will start showing a temperature and DC current dependency is partially related to the point at which the CNT loading stops being sufficient to generate a continuous conductive network. However, the formation of a conductive network could also be greatly affected by fabrication parameters and the state of dispersion of the filler in the insulating matrix [14]. Thus, explaining the existence of reports in which the reduction in resistance at higher temperatures is also detected on samples that have larger filler loadings, such as the CNT-polyether ether ketone (PEEK) thermoplastic composites, where resistivity was shown to decrease while increasing temperature from 20 degrees to 140 degrees Celsius for samples with 8, 9, and 10% CNTs [33].

While not depicted on the previous figures, testing extremely low CNT loading samples at high DC current or exposure to high temperature led to changes in electrical behavior of the samples (ca. a reduction in resistivity) over time. Since limited literature could be found related to the analysis of impacts of elevated current or temperature after these variables were no longer applied, that is, the effects reversibility/cyclability of the process, measurements were conducted with additional 0.014 wt% samples to determine if the observed shift in resistivity was a permanent or a temporary effect. All of the data, as discussed below in detail, indicate that for currents less than 3000 μA the changes in resistivity of the samples are only temporary (Section 3.3), while above 3000 uA (and more evident above 5000 μA) permanent changes (Section 3.4) are commonly evidenced by visible surface damage of the specimens and are expected to have an impact on the functionality of the composites.

### 3.3. Impact of Cycle Life/Aging on Composite Resistivity

**Figure 4:**
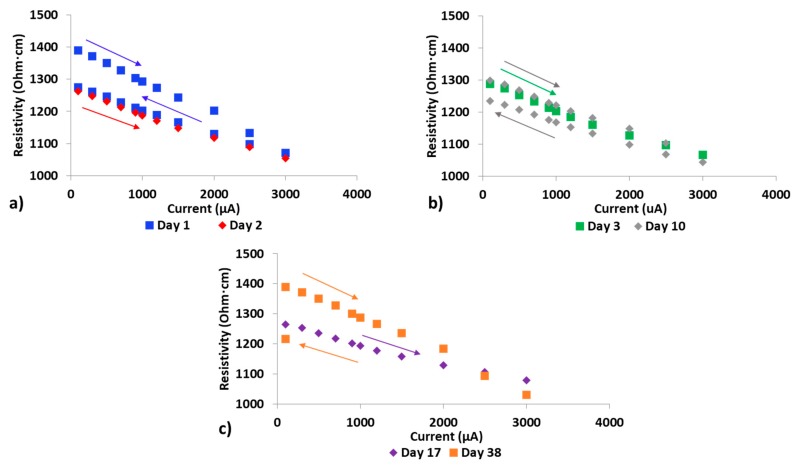
(**a**) 0.014 wt% sample subsequent day resistivity change; (**b**) 0.014 wt% sample one week resistivity change; (**c**) 0.014 wt% sample three week resistivity change.

A series of DC current trials increasing the current from 100–3000 μA were conducted on 0.014 wt% CNT composite samples. The tests were repeated after 24 and 48 h, after a week and after three weeks. Figure 4a–c shows graphs of resistivity vs. applied current for measurements taken one day apart (Figure 4a), after one week (Figure 4b), and after three weeks (Figure 4c). Resistivity values decreased in a similar way than the trends presented in Section 3.1.1. as current was increased up to 3000 μA. The resistivities recorded during the reduction of current showed only a partial upturn to original state, with final values below the initial ones. The changes in resistivity appear to be temporary, with the resistivity reduction remaining for less than one week and then typically reverting back to near original values.

On day one, resistivity at 100 μA was 1389 Ohm·cm at test commencement while at test conclusion, resistivity at 100 μA had dropped to 1274 Ohm·cm. On day two, the starting resistivity at 100 μA was 1263 Ohm·cm, which shows a retention of the change in resistivity from the prior day. Comparing day three and day ten, there is little difference between starting resistivity values at 100 μA, indicating that any additional lowering in resistivity that occurred when starting at 1288 Ohm·cm for 100 μA on day three was not retained for the one-week period. When comparing day 17 and day 38, the initial resistivity value at 100 μA on day 38 had returned to original value on day one (1389 Ohm·cm at 100 μA). However, as can be seen in Figure 4c, the temporary reduction in resistivity can be re-established by increasing the current to 3000 μA as the resistivity was 1217 Ohm·cm at 100 μA upon test completion.

The percentage change in resistivity when current is increased up to 3000 μA varies on different days with percentage decrease being ~23% on day one and typically a smaller percentage decrease on subsequent days until resistivity values have recovered to their near original values. However, it should be noted that the magnitude of these changes typically fall within the sample to sample variability of resistivity (typically in the 10^3^ to 10^4^ Ohm·cm range) observed in extremely low wt% epoxy-CNT composites.

Research by others with CNT-PEEK composites has shown indications of a change in resistivity that remained after removal of applied heat, however, the duration of this impact was not analyzed [33]. Research focused on rubber composites of ethylene propylene diene monomer and acrylonitrile butadiene with acetylene black showed resistivity impacts for these composites during heating and cooling cycles that are similar to those observed in current research [34]. Research done by Lasater et al. with CNT-vinyl ester composites and CNT concentrations between 0.1 and 1.0 wt% subjected those composites to nine thermal cycles from 25 to 165 degrees Celsius and showed that the 0.1 wt% sample had a slight decrease in resistance over the series of cycles whereas all others had an overall increase in resistance over the series of cycles [35]. Thus, similar observations to the ones presented in Figure 4 have been reported, however, retention of resistance values over time after conclusion of thermal cycling has not been thoroughly explored.

**Figure 5:**
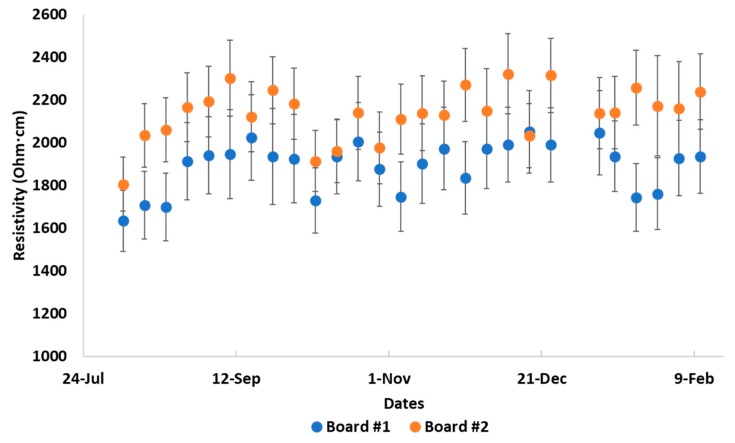
0.014 wt% sample long term resistivity at standard atmosphere and temperature.

An additional consideration to be accounted for when trying to explain changes in resistivity over time relates to the resistivity changes due to aging. To assess this impact, a set of two 0.014 wt% boards were produced and left inside a storage box exposed to atmospheric conditions. Resistivity readings at 100 μA were taken approximately once weekly for a period of over six months, generating the data presented in Figure 5. Error bars represent standard deviation for the eight measurements performed on each board.

Resistivity readings appear cyclical over time with a slight upward trend and a variability of ~15% from an average value over the course of the measurements. As can be seen in Figure 5, variability in resistivity values over time is not too different than variability observed between boards. Based on this data, the return of resistivity values to their original values before applying increased currents and/or heat may be somewhat influenced by the long-term effects of the epoxy-CNT composite being exposed to atmospheric conditions.

### 3.4. Conditions that Promote Surface Changes or Evidence of Incipient Failure

**Figure 6:**
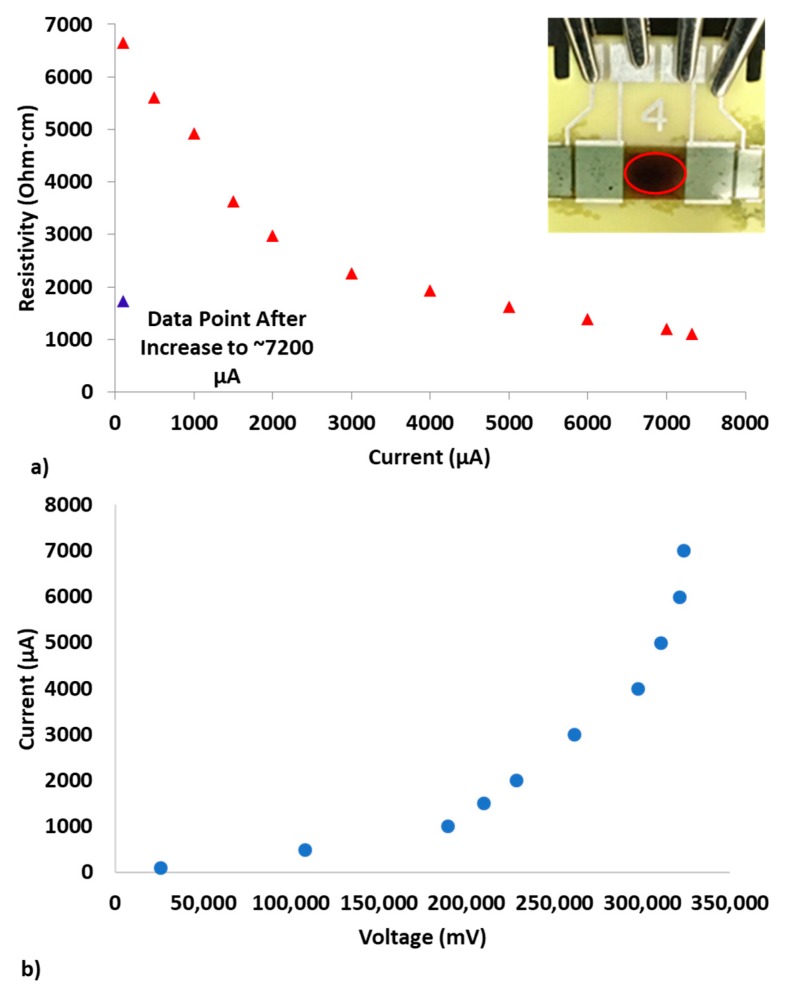
Current increase to above 7000 μA for 0.014 wt% sample; (**a**) resistivity vs. current; (**b**) current vs. voltage.

Following resistivity observations at DC currents up to 3000 μA, current was increased to near the maximum level of the source meter. As current was increased to higher levels (maximum current and temperature seen were 7320 μA and 226 degrees Celsius, respectively), the reduction in resistivity became more significant and clear non-ohmic behavior was observed, as seen in Figure 6a,b. The sample became discolored at a temperature of approximately 178 degrees Celsius, which corresponded to a current of ~5000 μA. This temperature corresponds closely with the maximum service temperature for EA9396 epoxy which is listed as 177 degrees Celsius [23]. Upon lowering temperature to 24 degrees Celsius and calculating resistivity at 100 μA, resistivity lowered from ~6650 Ohm·cm to ~1720 Ohm·cm.

The non-ohmic behavior of samples with extremely low loadings, like the one presented in Figure 6a,b for 0.014 wt% epoxy-CNTs, becomes more evident when higher currents are applied. This non-linear characteristic is also reported based on the analysis and simulations for CNT nanocomposites near a percolation threshold [36]. Additionally, polyvinyl butyral and polydimethylsiloxane CNT composites with higher loadings over a narrower voltage range and with higher CNT loading percentages have been reported to have non-linear resistance characteristics [37].

While the sample presented in Figure 6a, only became discolored, other samples began smoking and burning at similar currents. This observation is related to resistivity variability among samples and the temperatures that result from the application of high current. While the sample appearance was permanently changed, further analysis to examine which other properties suffer irreversible modification will ensue.

**Figure 7:**
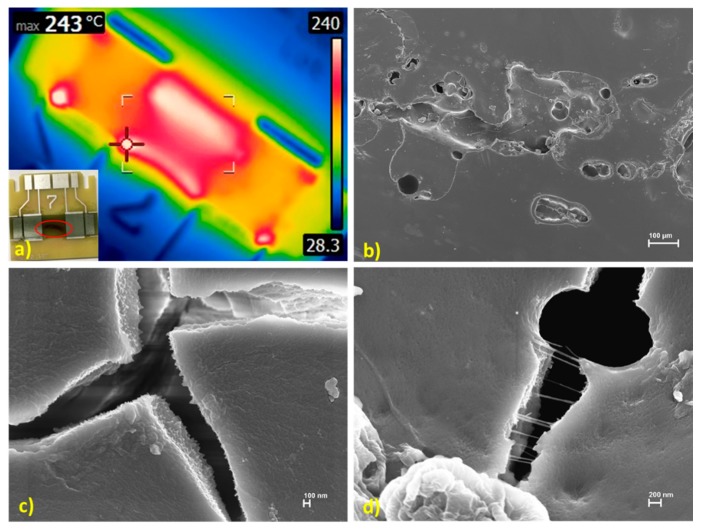
Images of 0.014 wt% sample taken to point of visible failure of epoxy-CNT composite. (**a**) FLIR and photo image; (**b**) SEM image of failure area; (**c**) SEM image of failure area location devoid of CNTs; and (**d**) SEM image of failure area location with CNTs.

A sample FLIR image at the point of blistering and cracking can be seen in Figure 7a. The failure area appeared as a dark red line with small cracks when viewed with the naked eye, while post-failure analysis in the SEM showed the damaged areas as consisting of blistering and bubbling of the epoxy-CNT composite (Figure 7b). An examination of the damaged areas showed many of the cracks and failure points as being devoid of CNTs (Figure 7c) whereas some of the smaller cracks had significant population of CNTs that appeared to be attempting to hold the epoxy matrix together (Figure 7d).

The images of the damaged epoxy-CNT composite areas illustrate that CNTs are concentrated differently throughout the composite and that the CNTs appear to play a role in maintaining the epoxy-CNT composite structure when blistering starts to occur at high temperatures/currents. While several studies report epoxy-CNT composite mechanical failures [38,39,40], literature related to epoxy-CNT conductive composite damage at high temperatures or high currents appears limited to environmental and safety studies [41]. An understanding of these impacts is necessary to define any operational limits for extremely low loading epoxy-CNT composites when used in various applications that will impose challenging temperature or current conditions.

The trends seem to indicate that with the lowered resistivities observed, the application of these formulations for ESD or EMI is not compromised unless the currents or temperatures imposed promote physical damage or negatively impact the mechanical properties.

### 3.5. Mechanisms Responsible for the Electrical Properties Trends Observed

In order to better understand the observed changes in resistivity, under varying experimental conditions, diverse hypothesis for the conduction mechanisms were examined and compared/contrasted with the literature. In extremely low loading epoxy-CNT composites, CNTs do not form a completely connected network since CNT loading is not high enough to support the complete conductive pathways through the material. As a result, conduction in the composite is a result of electron transport in the CNTs and via electron tunneling where gaps between CNTs exist. The following mechanisms that could affect the electron transport/tunneling at diverse currents and/or elevated temperature below the point of visible damage, were considered (a) thermal expansion differences among filler and matrix, (b) temperature dependent properties of the matrix, such as transition temperatures and viscosity, (c) thermal activation of charge carriers at elevated temperatures/thermal fluctuation induced tunneling, and (d) atmosphere/gas interactions.

Thermal expansion differences between CNTs and filler material have been assessed to separate the already connected CNTs or cause a larger gap distance between non-connected CNTs and hence a greater tunneling distance for fillers and a concomitant increase in resistance [30,42]. Specifically, Li et al. reported an increase in impedance for MWCNT/polyvinylidene fluoride (PVDF) composites with 4% and 6% vol CNTs as temperature is increased to near the composite melting temperature and assess that the reason is due to separation of filler material [42]. This trend is the opposite impact of observations made in the current work; however, it is noted that the filler loading in their case appears to exceed the loadings at which a complete conductive network will form.

At elevated temperatures above the melting temperature (or glass transition temperature) of polymers, a different effect is proposed in literature. Specifically, Li et al. report that when temperature is increased to around the melting temperature, and the polymer matrix becomes more fluid, CNTs become more mobile and more likely to form connections and lessen the gaps resulting in impedance decreasing for MWCNT/PVDF composites [42]. In current work, the decrease in resistivity occurs as soon as the temperature is increased and not near a point of phase change for the composite. This implies that epoxy softening is likely not the reason for the resistivity decrease seen in the extremely low loading epoxy-CNT composites of this study.

An increase in temperature could result in carrier thermal activation and overcoming of the barrier between filler materials which would result in a decrease in resistance. This thermal fluctuation induced tunneling [43] or hopping is proposed as the reason for decreases in impedance for epoxy-CNT composites with 0.5 to 1.0 wt% CNTs by Sanli [31] and as the dominant mechanisms for epoxy-CNT composites with 0.05 to 0.5 wt% by Shen et al. [30]. While this mechanism certainly could aid in explaining the resistivity decreases observed with increasing temperature, this mechanism would likely be reversible at the time at which heat is removed. As our observations show that resistivity remains lowered for a period of time after removal of elevated temperatures, this mechanism alone cannot fully explain the resistivity changes we observed or those observed by others [33,35]. Similar observations to the ones found herein were modeled by Kovacs et al. who proposed two types of percolation thresholds can coexist in an insulator–conductor-system, the higher one attributed to a static and the lower one to a kinetic network formation process [44]. “Kinetic” refers to the impact of stirring prior to epoxy hardening on flocculation. Moreover, those authors agree with our assessment, indicating that attention has to be paid to the diverse regions/loading regimes when modeling percolation behavior in these systems. In addition to our observed change in resistivity that remained for a period of days, Mohiuddin et al. observed in CNT-PEEK composites, albeit with much higher loadings (8, 9, and 10% CNT loading), a decrease in resistance for subsequent measurements following a temperature increase [33]. This resistance decrease was attributed to “…irreversible changes in the conducting networks caused by the initial heating process which induces some residual conductivity [33].” While some change in the conducting network has occurred, for our particular extremely low loading epoxy-CNT composites, the changes do not appear to be irreversible.

The interaction with gases in experimental environment should be also considered as a possible reason for the return of resistivity to near original values after removal of heat. While we observed fluctuations in resistivity values for a sample left exposed to atmospheric conditions over time, additional analysis is warranted to determine the extent of their impact.

In addition to the above discussion on temperature impacts, a simple model is considered for extremely low loading epoxy-CNT composites at ambient temperature. Percolation theory predicts at some loading there ceases to be a complete conductive path in composites. Once there is a “break” in the conductive path, a dramatic increase in resistivity is predicted, in agreement with the observation. Yet, it is observed that even after the sharp break, several orders of magnitude, in resistivity of the composite at extremely low loadings, the net resistivity for DC measurements is still eleven or twelve orders of magnitude lower than the epoxy matrix material. This highlights the clear role that CNTs play. A simple model, similar to that discussed by Sandler et al. [21] and the more complex model presented by Devivo et al. [45], for the observed resistivity behavior is presented: Electrically, the composite can be modelled as a system of parallel and series resistors and capacitors (matrix), connected by wires (CNT). At low frequency/DC conditions, the capacitive component naturally acts as an infinite resistor or open circuit. The length of the wires/CNT and the size of the resistors/capacitors (gaps) between CNTs are distributed over a broad range. In such a system, the net resistivity can be correlated with the net length of the gaps between wires. It is reasonable to suggest that at extremely low loadings, CNTs almost touch in some places and thus the net gap is a small fraction of the total length. Although the resistance of any gap is the same as that for the raw matrix material, the normalized resistance, expressed in units of Ohms/length, is far less because the resistor’s “length” is in fact a fraction of the total sample length. Moreover, in electric circuit theory, for any system of parallel resistors, the net resistance is always lower than the resistance of the lowest individual resistor. Thus, in this model the net resistance is predicted to be lower than that of the smallest gap in the system, however, this resistance will still be orders of magnitude greater than that of the CNTs. At sufficiently high frequency, the capacitive component with CNTs acting as conductive electrodes and matrix as the dielectric play a significant role. In this case, the path of least resistance will be through the capacitive component resulting in a decrease in resistivity at higher frequencies. The implication of this model is that the composite could potentially act as a high pass filter. This type of model is a simplified explanation for observed behavior and it must be realized that the extremely low loading epoxy-CNT composites consist of an infinite number of potential conduction paths comprising an infinite number of equivalent circuit components determined by the CNT connections and gaps between CNTs.

Identifying the role that each mechanism plays in the electrical properties of extremely low loading CNT composites and realizing an effective and straightforward model are challenging. Changes in resistivity with varying temperatures for CNT composites are impacted by CNT loading, matrix properties, changes to tunneling conduction at different temperatures, conductive properties of CNTs, contact resistance between CNTs, and other factors as demonstrated in this research and in others [15,30,31,33,35,42,44,46,47].

## 4. Conclusions

Based on the observed resistivity values, extremely low loading epoxy-CNT composites show significant promise for use in ESD and EMI applications. The potential dynamic nature of these applications will likely subject composites to varying temperature and current conditions, hence an understanding of these impacts is required. The presented work illustrates the impact of current and temperature on these composites while at the same time showing that the changes in resistivity values are less significant than resistivity variability introduced during the composite production.

This study illustrates that the resistivity of extremely low loading epoxy-CNT composites decreases during the application of elevated direct current and/or temperature and remains lowered for a period of days before returning to near original values. An analysis of AC effects shows that at different CNT loadings from 0.014 to 1.0 wt%, AC impedance values are nearly constant from 10 Hz to 10 kHz and correspond to DC resistance values while observed impedance changes at higher frequencies, suggesting their potential for use as high pass filters and warrant further analysis. A brief look at aging effects under normal atmospheric conditions shows resistivity value variability of approximately ~15% over a period of months which might be related to environmental effects. Samples taken to maximum epoxy service temperature or above, show permanent visible discoloration and blistering. Analysis of the damaged sections shows that areas devoid of CNTs are more likely to be damaged. A review of conduction mechanisms show that the observed trends are challenging to model, but are likely influenced by both matrix and CNT properties, thermal fluctuation induced tunneling and atmosphere interactions. Finally, a brief look at a model based on resistive and capacitive components provides a reasonable approach to understanding the electrical behavior of the extremely low loading epoxy-CNT composites at near ambient temperature.

This paper presents a detailed analysis of the electrical properties for extremely low loading epoxy-CNT composites and identifies the significant variables that will enable or limit their use in cutting edge technologies. The identified resistivity values and characteristics of these particular composites, even after exposure to higher currents and temperatures, are unique and highlight the need for further exploration.
